# Selective breeding can contribute to bovine tuberculosis control and eradication

**DOI:** 10.1186/s13620-023-00250-z

**Published:** 2023-08-24

**Authors:** Georgios Banos

**Affiliations:** Scotland’s Rural College (SRUC), Department of Animal and Veterinary Sciences, Easter Bush, Midlothian, EH25 9RG UK

**Keywords:** Bovine Tuberculosis, Cattle, Genetics, Genomics, Selective breeding

## Abstract

Bovine tuberculosis (bTB) persists in many countries having a significant impact on public health and livestock industry finances. The incidence and prevalence of new cases in parts of the UK and elsewhere over the past decades warrant intensified efforts towards achieving Officially Tuberculosis Free (OTF) status in the respective regions. Genetic selection aiming to identify and remove inherently susceptible animals from breeding has been proposed as an additional measure in ongoing programmes towards controlling the disease. The presence of genetic variation among individual animals in their capacity to respond to *Mycobacterium bovis* exposure has been documented and heritability estimates of 0.06-0.18 have been reported. Despite their moderate magnitude, these estimates suggest that host resistance to bTB is amenable to improvement with selective breeding. Although relatively slow, genetic progress can be constant, cumulative and permanent, thereby complementing ongoing disease control measures. Importantly, mostly no antagonistic genetic correlations have been found between bTB resistance and other animal traits suggesting that carefully incorporating the former in breeding decisions should not adversely affect bovine productivity. Simulation studies have demonstrated the potential impact of genetic selection on reducing the probability of a breakdown to occur or the duration and severity of a breakdown that has already been declared. Furthermore, research on the bovine genome has identified multiple genomic markers and genes associated with bTB resistance. Nevertheless, the combined outcomes of these studies suggest that host resistance to bTB is a complex, polygenic trait, with no single gene alone explaining the inherent differences between resistant and susceptible animals. Such results support the development of accurate genomic breeding values that duly capture the collective effect of multiple genes to underpin selective breeding programmes. In addition to improving host resistance to bTB, scientists and practitioners have considered the possibility of reducing host infectivity. Ongoing studies have suggested the presence of genetic variation for infectivity and confirmed that bTB eradication would be accelerated if selective breeding considered both host resistance and infectivity traits. In conclusion, research activity on bTB genetics has generated knowledge and insights to support selective breeding as an additional measure towards controlling and eradicating the disease.

## Introduction

Tuberculosis is a key disease with multiple pathogen and host species involved in transmission and consistently ranks in the top five most significant diseases worldwide [1]. According to the World Health Organisation’s “End TB strategy” new tuberculosis cases and deaths worldwide must be reduced by 90% and 95%, respectively, by the end of the 2016-2035 period [2]. The ambitiousness of these goals combined with the fact that about one third of the time has already elapsed set the stage for a considerable amount of intensified activity and attention, at multiple levels, to all Mycobacterium infections, including *Mycobacterium bovis (M. bovis)*, the key pathogen agent responsible for bovine tuberculosis.

Bovine tuberculosis (bTB) presents a global problem to the industry. As a zoonotic disease, bTB receives due attention as a public health, financial, and political issue. As such, monitoring, control, and eradication programmes have been established in multiple afflicted countries and regions.

In the United Kingdom (UK), bTB is endemic and costly to the tune of £175-200 million annually [3, 4]. The disease has returned and consolidated its presence in parts of Great Britain and Northern Ireland over the past 30-40 years. The key policy in place at the moment is national surveillance based mostly on animal reaction to the single intradermal comparative cervical tuberculin (SICCT) test, commonly known as the “skin test”, culling of positive animals, post-mortem examination and confirmation, and declaration of a bTB breakdown in the herd where the positive animal(s) was found combined with movement restrictions. Scotland has been the only Officially Tuberculosis Free (OTF) nation in the UK since 2009, while the goal is for England to achieve this status by 2038 followed by the other regions. To achieve OTF status, the percentage of new breakdowns must not exceed 0.1% per year for six consecutive years [5]. According to 2009 data, 5-6% of herds in the non-OTF parts of the UK were affected [6] and the figure did not improve until recently [7]. However, the number of new breakdowns in England in the 12-month period to March 2022 was 2,892, down 8.80% from the previous 12-month period ending in March 2021 [7]. A similar reduction was observed in the number of cattle slaughtered due to a bTB incident in the corresponding time period. An average annual decreasing trend of about 8% in new herd incidents was also observed in the previous four 12-month periods. The challenge now is not only to maintain but, importantly, accelerate this downwards trend over the next decades to ensure the OTF target is met.

Selective breeding has been proposed as an additional possible means to control bTB, thereby contributing to the ongoing monitoring and eradication programmes. The main premise is that individual animals exposed to the same pathogenic conditions responsible for a disease react and respond differently: some animals remain healthy, others become a little sick, others more so, and some actually die. This variation is partly attributed to genetic factors manifested in protein-coding genes and/or regulatory sequence elements that render certain animals inherently more resistant or susceptible to the disease than others. In the case of bTB, identifying animals that carry genetic variants contributing to infection resistance and selecting them for breeding can promote the overall population resistance and contribute to bTB control. In a similar but more conservative scenario, individual animals bearing genetic variants that render them susceptible to the disease are identified and removed from the breeding process, thereby removing said variants from the subsequent generations. The potential of selective breeding in bTB control is reflected in a relatively recent strategy review commissioned by the UK government, led by Sir Charles Godfray of the University of Oxford [8] where a relevant excerpt reads as follows: ”*There is evidence that cattle vary genetically in their susceptibility to bovine TB and, once infected, there may be genetic variation in their propensity to spread the infection to other animals. It is thus possible to select for better disease susceptibility and transmission traits as part of cattle breeding programmes.*” [8].

The present review discusses the genetic component of variation in host response to bTB infection and demonstrates how this variation may be applied in selective breeding programmes towards enhancing cattle resistance to bTB. Results from relevant studies are summarised showing and quantifying the potential impact on disease control and eradication. Further to disease resistance, the role of genetics in reducing animal infectivity regarding bTB is briefly discussed.

### Genetic variation among animals for resistance to bTB

The genetic basis of host resistance and susceptibility to bTB infection was addressed in a 2010 review [9] and potential future benefits were already acknowledged at the time. Multiple studies were subsequently conducted and quantified the genetic variation for bTB in different cattle populations and countries, using a variety of statistical models and trait definitions, and reported heritability estimates that range mostly between 0.06 and 0.18 [10–15]. The majority of studies were based on large amounts of field data combined with extensive animal pedigree information. Although different datasets and trait definitions were considered in these studies, all phenotypes related to animal response to bTB infection and reflected their susceptibility or resistance to the disease. The range of reported heritability estimates means that 6-18% of the differences observed among individual animals in their response to the pathogen are attributed to heritable genetic factors. In two studies based on the UK national bTB surveillance data, heritability of bTB resistance was 0.09-0.12 in dairy cattle [14] and 0.09-0.11 in beef cattle (author’s unpublished results, 2020).

More recent studies included animal genomic data based on genotyping with genome-wide DNA arrays in the calculations and reported higher heritability estimates of 0.21-0.27 [16–19]. The advantage of using such data is that they more accurately capture finer details of the genetic background dictating animal resistance to the disease. The caveat is that the amount of genomic data is usually smaller compared to the size of pedigree data on which conventional genetic studies are based; heritability estimates also tend to be sometimes inflated. Fortunately, animal genotyping costs are decreasing rapidly, and the number of individual animals genotyped has been notably increasing in many breeding programmes worldwide, leading to improved accuracy of the genomic estimates. More discussion on genome-wide data in bTB studies is provided in a later section of this review.

The overall magnitude of heritability reported so far may seem moderately low but for most animal traits of interest estimates normally do not exceed 0.50. Heritability of health-related traits is known to be generally modest to low, usually less than 0.15. Nevertheless, the genetic variation manifested in the above heritability estimates for bTB resistance is enough to achieve progress with selective breeding. The latter has been decisively demonstrated for other cattle health traits with similar or even lower heritability values such as clinical mastitis, lameness, and metabolic diseases, which now feature prominently in selective breeding programmes worldwide [20].

In conclusion, there is confidence that genetic variation in cattle for bTB resistance is indeed sufficient to identify animals that are inherently more resistant than others to *M. Bovis* infection. Selective breeding programmes would then need appropriate genetic evaluation systems and tools in place to properly identify the inherently resistant and susceptible animals in order to inform breeding decisions that will underpin enhanced resistance in the subsequent generations.

### Genetic evaluations for bTB resistance

Research conducted on bTB surveillance data in the UK led to the development of a national genetic evaluation of dairy cattle for bTB resistance [14]. This system estimates the genetic merit or breeding value of each individual dairy bovine reared in the country for resistance to the infection. Supported by the Agriculture and Horticulture Development Board, the national farm levy body, the genetic evaluation project collated relevant data from different national databases, including: bTB records from surveillance in Great Britain and Northern Ireland, animal location and movement data, production history, and extensive animal pedigree information.

The study first combined SICCT test and post-mortem data, namely records on bTB lesions and *M. Bovis* culture results, to define the health status of each animal, which would constitute their individual phenotype for bTB infection. Furthermore, probabilities of animal infection at different stages of a breakdown were assigned to each animal that eventually tested positive to the SICCT test [14]. These probabilities were based on the sensitivity estimates of the test as reported in the literature. Animals that remained healthy throughout the epidemic were included in the data as controls.

A genetic model was developed to analyse these bTB phenotypes [14]. The model was subsequently expanded and, in its final form, accounts for multiple non-genetic factors that are known to affect the trait, such as the location and duration of the breakdown, the calendar year and month the breakdown started, the herd size, the age and lactation number of the cow, and factors such as heterosis and recombination, since the genetic evaluation combines data from multiple dairy breeds and must account for breed composition and potential crossbreeding.

Further to non-genetic effects, the model includes an animal additive genetic effect that, after data analysis, provides an estimate of the genetic merit of each individual animal for resistance to bTB that accounts for all other sources of systematic variation fitted in the model. Here, although the phenotypic data pertain only to milking cows, breeding values are also estimated for their sires, through the pedigree connections that are included in the data analysis. Actually, the sire breeding values are more accurate than those of individual cows on the account of the former being associated with multiple progeny with records in the system. In a validation exercise, the correlation of the sire estimated breeding values with the proportion of infected milking daughters was -0.68, suggesting that sires with highly positive values of genetic merit are associated with reduced infection rate in their progeny [14]. Similarly, the daughters of the 20 genetically most resistant sires are 21% less likely to become infected that those of the 20 most susceptible sires [14].

Importantly, the correlation between genetic evaluations for bTB resistance and other animal traits is mostly zero, suggesting that selective breeding to enhance animal resistance to the disease would not compromise improvement in the other traits [14]. In fact, moderate positive desirable correlations have been reported of bTB with lifespan and the Profitable Lifetime Index (£PLI), the overall dairy selection index in the UK that combines the economically important animal traits in one single value [14]. This result consolidates the expectation that enhancing the inherent cattle resistance to bTB would have favourable effects on animal longevity, with the associated benefits of enriched animal welfare, reduced waste, and improved environmental footprint.

A subsequent study expanded the bTB genetic evaluations to include genome-wide marker data from animal genotyping using DNA arrays [21]. Different models of genomic analysis were investigated and the single-step Best Linear Unbiased Prediction (BLUP) method [22, 23], which allows the simultaneous combination of all phenotypic, genomic and pedigree data available, resulted in the most accurate estimates of animal genetic merit for bTB resistance [21]. Results from this type of analysis would facilitate the identification and selection of young breeding animals early in life, thereby delivering faster benefits of selective breeding in bTB control and eradication programmes.

Research on the genetics of bTB resistance in dairy cattle has led to the development of the official UK national genetic evaluation, run since 2016 at the Edinburgh Genetic Evaluation Services on behalf of the Agriculture and Horticulture Development Board. The genetic evaluation outcome, the actual selection index, has been termed “TB Advantage” [24] and is now available for dairy sires of all breeds with progeny (milking daughters) in the national bTB surveillance programme. TB Advantage is expressed on a scale normally ranging from -4 to +4, where, for every +1 point in the index, 1% fewer daughters are expected to become infected during a bTB breakdown. This information allows dairy farmers to focus on the most positive or remove the most negative TB Advantage bulls from the list of potential sires selected to artificially inseminate their cows.

Another research project investigated the feasibility of a genetic evaluation for bTB resistance in UK beef cattle. Data analyses were conducted on 437,235 animals, progeny of 36,790 sires from 53 beef breeds. These data pertained to 8,648 bTB breakdowns where 7.3% of the animals were infected (author’s unpublished results, 2020). Significant genetic variation was detected for bTB resistance and was manifested in variation in the resulting estimated breeding values of the sires, thereby demonstrating the feasibility to distinguish breeding animals expected to produce bTB resistant from susceptible progeny. As was the case with dairy breeds, correlations of these breeding values with beef traits such as animal growth, carcass characteristics and lifespan were either negligible or moderately favourable, suggesting that genetic selection to enhance bTB resistance in beef cattle would not compromise the current breeding goal. Incorporation of bTB in the official national genetic evaluation process of beef cattle is imminent.

Genetic evaluations for bTB resistance are also available in the Republic of Ireland [25] and are offered as tools for genetic selection to complement the on-going national bTB control programme towards accelerating the disease eradication process. These evaluations are based on relevant population genetic research conducted on Irish bTB surveillance data [13, 15] that demonstrated the presence of sufficient genetic variation and the feasibility of accurate genetic evaluations for the trait. The actual evaluations are run since 2019 by the Irish Cattle Breeding Federation for both dairy and beef breeds in the country [25]. Estimated breeding values are expressed as the predicted proportion of infected progeny of a sire. In this regard, contrary to the UK genetic evaluation, lower values are desirable. For example, when two sires with estimated breeding values of 5% and 10% are assessed, 5% fewer progeny of the first sire are expected to be diagnosed as infected compared to the second.

Despite being expressed on distinctly different scales in Ireland and the UK, estimated breeding values for resistance to bTB offer essentially the same information, namely, they identify and separate genetically resistant and susceptible individuals. Thus, they have the same potential utility in supporting selective breeding programmes in the respective countries. The key is in the interpretation and proper use.

These genetic evaluations are now well tested and validated nationally, so that combination and a joint analysis of data from the two countries to produce across-country estimated breeding values for bTB become a realistic option. Experience with the international genetic evaluations provided by the International Bull Evaluation Service (Interbull) [26] since 1994 for an array of other bovine traits has been very positive. It is clearly possible to combine data from different countries and populations with good genetic links, account for differences in the national definitions and systems, analyse jointly, and then express the estimated breeding values of all selection candidates available internationally on the national scale used in each country. In addition to enhancing the accuracy of the genetic evaluation and broadening the selection pool for the farmers, outcomes facilitate trade across the border, streamline the use of bovine genetics available globally, and promote collaboration and harmonisation of practices in the participating countries.

### Genomic architecture of cattle resistance to bTB

The advent and broad implementation of bovine genome-wide DNA arrays (chips) based on tens or hundreds of thousands of Single Nucleotide Polymorphism (SNP) markers have enabled research that cast closer insights on genomic regions and genes associated with animal traits of interest. The key principle is that these SNPs are within or in high linkage disequilibrium with genes and genomic regulatory regions that control important animal traits. This approach has found widespread use in livestock breeding programmes [27].

Multiple genomic association studies using data from the above mentioned DNA arrays have been conducted on bTB resistance in various bovine populations in different countries [15, 16, 18, 19, 28–31]. The majority of the studies reported single markers with relatively modest effects on the trait. Furthermore, mainly different markers were found associated with cattle resistance to bTB across these studies, partly reflecting differences among populations, trait definitions, DNA array specifications, and models of statistical analysis. Nevertheless, the overall picture is that of a trait that is not being controlled by only a single or a few genes.

In addition, a few regional heritability mapping studies have been conducted using genome-wide SNP data [16, 18, 32]. Some of these studies revealed genomic regions associated with bTB resistance that included significant SNPs identified in the genomic association, but others were not necessarily consistent with single marker results on the same data. In the latter case, these would be genomic regions that harbour multiple markers and genes, each with a small undetectable effect, but combined with a more substantial and discernible effect on bTB resistance.

Using publicly available bovine genome assemblies, several annotated genes were identified neighbouring SNPs or being located within genomic regions associated with bTB traits in the aforementioned studies. Further studies more closely examined specific candidate genes affecting the host’s resistance or susceptibility to the disease [33–38]. These studies offered novel insights into the function and role of the individual genes in networks and pathways that are linked with the animal’s capacity to fight off infection.

Table 1 summarises the genes that have been reportedly associated with cattle resistance to bTB in different genomic association, regional heritability mapping, and candidate gene studies. The collective outcome of these studies is that cattle resistance to bTB is a largely polygenic trait dictated by multiple genes across the bovine genome. Therefore, selective breeding based on genetic and genomic evaluations as previously discussed would be the best approach towards genetic improvement of the trait. Furthermore, identification of single markers and genomic regions with noticeable effects may inform the genetic evaluation process, with due emphasis being placed on these markers and regions resulting in an increase in the accuracy of the estimate of the genetic merit of individual animals for resistance to bTB.

**Table 1:** Genes associated with host resistance to bovine tuberculosis in different research studies in the past 10 years demonstrate the polygenic nature of the trait

Reference	[28]	[16]	[29]	[33]	[30]	[34]	[35]	[18]	[32]	[15]	[31]	[36]	[19]	[37]	[38]
Year	2012	2014	2015	2015	2016	2016	2017	2017	2017	2019	2019	2020	2020	2020	2021
Country^a^, Breed^b^	IE, HO	IE, HO	ET, HAX	TW, HO	IE, HO	TW, HO	CN, HO	UK, HO	UK, HO	IE. ML1	MX, HO	IN, MLX	CM, FX	UK/PK, ML2	CN, HO
No. animals	986	1,151	502	150	841	164	232	804	1,966	14,778	375	245	212	45	283
Approach^c^	GWAS	GWAS, RHM	GWAS	SCG	GWAS	SCG	SCG	GWAS, RHM	RHM	GWAS	GWAS	SCG	GWAS	SCG	SCG
**Chromosome**															
1					*KALRN*						*CD80, TIGIT, NAALADL2*				
2		*MYO3B*		*SLC11A1*			*SLC11A1*	*PARD3B*			*DNER*			*SLC11A1*	
3											*CTSS, FCGR1A, TRIM33*				
4					*DPP6*										
5											*CD163*				
6			*TLR1, TLR6, TLR10, RHOH*							*ANTXR2*		*TLR1*			
10					*AP3B1*					*JMY*					
13		*PTPRT*									*MACROD2, KIF16B*				
15										*OR52E5, ENSBTAG00000023125, ENSBTAG00000047246, ENSBTAG00000048223, ENSBTAG00000023912*					
16										*SRGAP2, RASSF5*					
17										*CLGN*					
19															*NOS2A*
22	*SLC6A6*								*RAF1, MKRN2, MKRN2OS, TSEN2, PPARG, MIR2373*						
23					*FKBP5*	*TNFα*		*RNF144B*	*LRRC1, LOC104975626, KLHL31, GCLC, DSB, BOLA-DYA, BOLA-DYB, BOLA-DOB, LOC100140517, MLIP, LOC104975626*	*RCAN2*	*HFE*	*TNFα*			
25											*IL21R*				
29											*ANO9, SIGIRR*		*PICALM*		

^a^Country of study: *IE* Ireland, *ET* Ethiopia, *TW* Taiwan, *CN* China, *UK* United Kingdom, *MX* Mexico, *IN* India, *CM* Cameroon, *PK* Pakistan

^b^Bovine breed of study: *HO* Holstein, *HAX* HO x Arsi Zebu cross, *ML1* Multiple beef and dairy breeds, *MLX* Multiple indigenous breeds and crosses, *FX* Fulani and crosses, *ML2* HO, Brown Swiss, Sahiwal

^c^Data analysis method: *GWAS* Genome-Wide Association Study, *RHM* Regional Heritability Mapping, *SCG* Single Candidate Gene

### Potential impact of selective breeding on the disease dynamics

Genetic improvement with selective breeding is a long-term process and benefits become visible in future generations. From the moment the genetically most resistant breeding animals are identified and mated, it would take at least one generation to see the effect in their progeny. The process will continue in the subsequent generation and then in the following one and so on, and benefits gradually accumulate. The process is slow, but the effects are permanent and cumulative. The benefits of selective breeding have been observed in multiple livestock traits not least those associated with animal production, health, fertility, and longevity over the past century and have been duly documented [20].

Presence of heritable genetic variation and the feasibility of harnessing it in the estimation of accurate genetic evaluations of animals, as discussed above, may underpin selective breeding practices aiming to enhance resistance to bTB and contribute to the overall control and eventual eradication of the disease. In a generational context, these selection tools have become available relatively recently and the full effects are expected to materialise in due course in the subsequent generations.

In the meantime, simulation studies have been conducted to predict and quantify the long-term effect of selective breeding for enhanced animal resistance or reduced susceptibility to bTB. In one such study, animal data were simulated reflecting the current bTB dynamics in the UK regarding disease prevalence, and size and duration of bTB breakdowns [39]. A model of disease transmission was implemented based on a sequence of stages, where the animal: (i) is at first susceptible but not infected, (ii) is then exposed to the pathogen, (iii) becomes infectious and (iv) is finally detected as infected with the SICCT test and, therefore, removed from the herd. This choice of model was based on empirical data showing that infected cattle may become infectious before entering the detectable stage [40]. The same level of genetic variation among individual animals in bTB resistance found in the real data analyses [14] was introduced in the simulations. Subsequently, various levels of selection emphasis on bTB resistance were examined: in the most intense selection scenario, the 10% genetically most resistant sires were selected to breed the next generation, whereas in the least intense case 70% were selected. The latter reflects a conservative approach where the 30% genetically most susceptible animals are removed from breeding. A baseline scenario of no selection was also examined for comparison. Twenty generations of selection were simulated, roughly corresponding to 80-100 years in a dairy cattle conventional breeding programme.

Results showed that any level of selective breeding would be associated with a faster ending of the epidemic compared to the scenario of no selection [39]. As expected, higher selection intensities had a stronger impact on the epidemic profile. The probability of a breakdown to occur would half after about 5 generations of intense selection of the top genetic merit sires. A more conservative approach based on avoiding the genetically most susceptible sires in breeding decisions would take about 12 generations to reduce the probability of a breakdown to one half. When a breakdown actually occurs, selective breeding would decrease its duration and severity manifested in the number of infected animals after the breakdown had been declared. After one generation of selection, duration and severity could decrease by 5-10% and 11-17%, respectively, dependent on the intensity of selection [39]. In practice, selection programmes may be adapted to the geographical risk levels, with actively intense selection applied in high-risk areas and moderate conservative practices elsewhere.

The above research was based on a model reflecting the “worst-case” scenario where an animal becomes infectious and starts transmitting the disease before it can be detected as infected and removed from the herd. Although this assertion is consistent with the suggestion that cattle with bTB lesions in the respiratory tract should be considered potential transmitters of the pathogen and sources of infection for other animals in the herd [41], previous non-genetic epidemiological studies on bTB typically assumed that infected animals usually react positively to the SICCT test and are removed from the herd before they become infectious [42, 43]. The two models are discussed in detail in [39]. Further genetic epidemiological studies reversing the order of the two stages so that infected animals were detectable before they became infectious demonstrated that the benefits of selective breeding were proportionally still the same compared to the no-selection option [39].

Key conclusions drawn for this research were that genetic selection could accelerate bTB eradication, as a complementary measure to already existing ones. Thus, selective breeding for enhanced animal resistance to bTB can contribute to herds and regions achieving their OTF goals faster.

### Genetic background of bTB infectivity

Further to improving animal resistance to bTB, a disease eradication programme would also benefit if the rate of transmission from one animal to another decreased. There is increasing evidence, albeit mostly empirical, that individual animals differ in their capacity to infect their herd-mates. The presence of inherently super-spreader individuals is now widely accepted [44] and identifying them early and removing from the breeding process would yield additional benefits. Selective breeding practices could then be expanded to harness this novel host genetic variation and contribute to decreasing infection rates.

Relevant genetic-epidemiological simulation studies have suggested that selective breeding for reduced infectivity together with enhanced resistance to bTB would lead to faster disease eradication than selecting only for enhanced resistance or, of course, not selecting at all [45, 46]. These simulation studies showed that the magnitude of the benefit would vary depending on selection intensities, the heritability of infectivity, and the genetic correlation between infectivity and resistance. Several selective breeding scenarios were assessed in these studies, and in all cases the epidemic risk decreased dramatically, when infectivity and resistance were considered together. For example, in one scenario assuming moderate selection intensity, trait heritability and prediction accuracy, the epidemic risk after five generations of selection decreased by 50% when selective breeding decisions considered infectivity [45]. In another scenario with similar assumed parameters, it took seven generations for the bTB epidemic to die out with selective breeding for reduced susceptibility and three generations when selective breeding aimed at reducing both animal susceptibility and infectivity [46].

However, the genetic basis of infectivity is still largely unknown. Defining and characterising the proper animal phenotype is a serious challenge. Work is currently being conducted on this topic and a recent study on dairy cattle data reported significant genetic variation among index cases regarding the number of secondary cases attributed to them [47]. Here an index case would be the first animal tested and confirmed bTB positive, thereby instigating the breakdown, and secondary cases would be the animals that tested positive in the first stages of the breakdown and assumed to have been infected by the index case. Thus, the trait may be considered as a proxy for animal infectiousness. Different models of analysis were considered and preliminary heritability estimates of 0.09-0.21 derived were of similar magnitude as the heritability estimates for bTB resistance discussed previously. Continuing work addresses refinements in the definition of animal infectivity and modelling improvements. Although more research is definitely warranted to properly define and dissect the genetic basis of infectivity and understand the potential for genetic improvement, these first results are encouraging.

### Novel bTB phenotypes

Availability of informative individual animal phenotypes for the traits of interest is the key and most crucial component of modern selective breeding programmes. This entails data of high quality that reflect the true biology of the animal as closely as possible, available in sufficient quantities to allow accurate calculations. Without said phenotypes, even the most advanced genomic technologies and the most elaborate statistical analysis models would have very little opportunity to demonstrate their utility.

In the case of bTB, SICCT test records combined with post-mortem visible lesions and *M. bovis* culture results constitute the phenotypes in current national genetic evaluations [24, 25]. Thanks to the ongoing national surveillance programmes, such data span multiple decades and cover all affected geographic regions in a country. The depth and amount of data in combination with the high specificity of the SICCT test render these phenotypes and resulting genetic evaluations valuable for selective breeding purposes.

Other diagnostics that may provide new animal phenotypes with improved sensitivity are worth consideration. Gamma-interferon assays have been long used for bTB diagnosis [48] often in combination with the SICCT test [49], especially in high-risk geographic regions. Nevertheless, the development of individual animal phenotypes for bTB based on gamma-interferon to complement the existing ones based on SICCT tests and post-mortem examination results and use in bovine selective breeding programmes has not been explored.

Alternative phenotyping approaches have been proposed in livestock improvement based on molecular traces in the milk of dairy cows that emanate from various animal physiological processes [50]. Mid-infrared (MIR) spectroscopy of milk samples may capture these traces in an effective, non-invasive, and non-interfering process. This has led to the accumulation of large amounts of MIR data in routine milk recording systems in many countries. Analysis of MIR data already provides useful proxy phenotypes for different animal traits associated with milk composition [51], cow body energy [52], pregnancy diagnosis [53], greenhouse gas emissions [54], and feed intake [55].

In a recent study, extensive cow milk MIR data were combined with bTB national surveillance records and machine learning was deployed to jointly analyse the data and deliver a prediction pipeline of the bTB status of individual animals from their MIR spectral profiles [56]. After optimisation of the training process, including data and network, the specificity, sensitivity and accuracy of predictions reached 0.94, 0.96 and 0.95, respectively [56]. These results lend confidence in the method to predict future bTB status of individual animals, thereby offering farmers a useful tool for early management decisions on cows likely to become infected. Furthermore, these predictions may constitute additional novel animal phenotypes for bTB, warranting genetic studies to determine their suitability for genetic evaluations and improvement with selective breeding.

### Closing remarks and outlook

Bovine tuberculosis is a complex issue, and an array of factors may determine whether an animal becomes infected, and a breakdown is declared. Complex problems require complex strategies and solutions, and indeed this is the case with ongoing bTB control and eradication programmes in different countries. Genetic selection of inherently resistant individuals that will breed enhanced natural resistance into the subsequent generations is proposed as an additional component within these programmes.

Further improvements in genomic evaluations are in scope with more targeted genotyping both in terms of animals (bulls and cows) and DNA arrays, and incorporation of identified genomic regions with significant effects on the trait in the calculation of breeding values. Decreasing DNA array genotyping costs, genotype imputation improvements, and new approaches such as genotyping by low pass sequencing combined with imputation provide useful opportunities in this regard.

As previously mentioned, the polygenicity of host resistance to *M. bovis* infection is now well accepted. Future genomic studies of DNA array and whole genome sequence data combined with post-genomic (transcriptional and translational) research will cast further insights into the complex architecture of the trait, including focus on the location and function of both protein-coding genes and regulatory elements [57]. Improvements in the functional annotation of the bovine genome [58] certainly facilitate such research activities, leading to enhanced precision and biological relevance of the genomic evaluation and selective breeding.

Beyond host resistance to *M. bovis* infection, understanding the genetics of infectivity at the individual animal level is only now starting and we have some way to go before being able to usefully inform genetic selection programmes aiming to decrease the rate of animal-to-animal transmission. Nevertheless, this area of scientific research and development is topical and warrants continuing attention and effort. Future bTB control programmes should be able to combine selective breeding for increased host resistance with reduced animal infectivity.

Furthermore, as has been experienced with the genetics of just about any important animal trait, multi-actor collaboration is a prerequisite to the successful development and implementation of selective breeding programmes. There is scope for continuing interdisciplinary research to understand why and how individual genes affect host traits and how host genetics interact with pathogen variation. Moreover, there are opportunities for across-country cooperation to facilitate international trade that is of key importance to the bovine industry. There are national genetic evaluation systems for bTB in place in at least the UK and Ireland and it is possible to pull data together and produce international genetic evaluations, as is the case with several dozens of other animal traits at the moment [26].

As already mentioned, selective breeding is a long-term process, it takes time to see the benefits, but it can work. Of course, this requires faith and good judgement, and everybody to be on board, not least farmers, practitioners, breeder associations, breeding companies, scientists, governments, and policy makers. Only then would the benefits discussed here truly materialise, contributing decisively to the control and eventual eradication of the disease.

## Data Availability

Not applicable.

## Abbreviations

bTB: Bovine Tuberculosis
OTF: Officially Tuberculosis Free
M. bovis: Mycobacterium bovis
UK: United Kingdom
SICCT: Single Intradermal Comparative Cervical Tuberculin
£PLI: Profitable Lifetime Index
BLUP: Best Linear Unbiased Prediction
Interbull: International Bull Evaluation Service
SNP: Single Nucleotide Polymorphism
MIR: Mid-infrared
